# Activated Carbon/Pectin Composite Enterosorbent for Human Protection from Intoxication with Xenobiotics Pb(II) and Sodium Diclofenac

**DOI:** 10.3390/molecules27072296

**Published:** 2022-04-01

**Authors:** Jakpar Jandosov, Mo Alavijeh, Shynggyskhan Sultakhan, Alzhan Baimenov, Maria Bernardo, Zuriyadda Sakipova, Seytkhan Azat, Svitlana Lyubchyk, Nurzhamal Zhylybayeva, Gulmira Naurzbayeva, Zulkhair Mansurov, Sergey Mikhalovsky, Dmitriy Berillo

**Affiliations:** 1 School of Pharmacy, Asfendiyarov Kazakh National Medical University, 94, Tole Bi Street, Almaty 050000, Kazakhstan; sakipova.z@kaznmu.kz (Z.S.); berillo.d@kaznmu.kz (D.B.); 2 Institute of Combustion Problems, 172, Bogenbay Batyr Street, Almaty 050000, Kazakhstan; shynggyskhan.1@gmail.com (S.S.); gumiw@mail.ru (G.N.); zmansurov@kaznu.kz (Z.M.); 3 Pharmidex Pharmaceutical Services, 14, Hanover Street, London W1S 1YH, UK; 4 Chemistry Division, Al-Farabi Kazakh National University, 71, Al-Farabi Avenue, Almaty 050040, Kazakhstan; alzhan.baimenov@nu.edu.kz (A.B.); seytkhan.azat@gmail.com (S.A.); nurzhamaljk@mail.ru (N.Z.); 5 National Laboratory Astana, Nazarbayev University, 53, Kabanbay Avenue, Nur-Sultan 010000, Kazakhstan; 6 LAQV/REQUIMTE, Departamento de Química (DQ), Faculdade de Ciências e Tecnologia (FCT), Universidade Nova de Lisboa (UNL), 2829-516 Caparica, Portugal; maria.b@fct.unl.pt (M.B.); s.lyubchyk@fct.unl.pt (S.L.); 7 Laboratory of Engineering Profile, Satbayev University, Almaty 050012, Kazakhstan; 8 ANAMAD Ltd., Sussex Innovation Centre Science Park Square, Falmer, Brighton BN1 9SB, UK; sergeymikhalovsky@gmail.com; 9 Chuiko Institute of Surface Chemistry, National Academy of Sciences of Ukraine, 17, General Naumov Street, 03164 Kyiv, Ukraine; 10 Atchabarov Research Institute of Fundamental and Applied Medicine, 94, Tole Bi Street, Almaty 050000, Kazakhstan

**Keywords:** diclofenac adsorption, lead adsorption, enterosorbent, pectin, porous carbon

## Abstract

The use of enterosorbents—materials which can be administered orally and eliminate toxic substances from the gastrointestinal tract (GIT) by sorption—offers an attractive complementary protection of humans against acute and chronic poisoning. In this study, we report the results of developing a microgranulated binary biomedical preparation for oral use. It was designed with a core-shell structure based on pectin with low degree of esterification as the core, and nanoporous activated carbon produced from rice husk, AC-RH, as the shell, designated as AC-RH@pectin. The adsorption properties of the synthesized materials were studied in aqueous solutions for the removal of lead (II) nitrate as a representative of toxic polyvalent metals and sodium diclofenac as an example of a medicinal drug. The composite enterosorbent demonstrated high adsorption capacity for both adsorbates studied. Adsorption kinetics of lead and diclofenac adsorption by AC-RH, pectin, and AC-RH@pectin, fitted well a pseudo-second-order model. According to the Langmuir adsorption isotherm model, the best fitted isotherm model, the maximum adsorption capacity, q_max_, of AC-RH@pectin for diclofenac and for lead (II) was 130.9 mg/g and 227.8 mg/g, respectively. Although q_max_ of AC-RH for diclofenac, 537.6 mg/g, and q_max_ of pectin for lead (II), 245.7 mg/g, were higher, the maximum adsorption capacity of AC-RH for lead (II), 52.7 mg/g, was much lower than that of the composite AC-RH@pectin and the adsorption capacity of pectin for diclofenac was negligible. Therefore, the composite material AC-RH@pectin demonstrated substantial efficiency of removing both species which potentially defines it as a more universal enterosorbent suitable for treating poisoning caused by substances of different chemical nature.

## 1. Introduction

Oral sorbents or enterosorbents have been used as a medical tool to treat acute and, more seldom, chronic poisoning of humans with toxic substances of exogenous and endogenous origin [1]. A variety of materials such as polysaccharides, activated carbon, inorganic oxides and minerals, porous synthetic polymers and others have shown ability to neutralise harmful effects of xenobiotics on human health by adsorbing them from the gastrointestinal tract (GIT). A multitude of enterosorbents reflects the large variety of the chemical nature and speciation of xenobiotics spanning from soluble compounds of radionuclides and heavy elements to molecular organic substances such as pesticides, drugs and biotoxins. Currently, none of the enterosorbent formulations has the capacity to remove efficiently toxic substances in the whole range of xenobiotics. Activated carbon (AC), for example, has high sorption capacity for small and medium size organic molecules but low affinity for metal ions, whereas sorbents containing a large number of ion exchange groups show significant sorption capacity for ionic species such as metal ions. Pectin biopolymers are natural polysaccharides which have unique biological and functional characteristics that determine their importance for plant biology and they are also widely used in food industry and cosmetics mainly as an emulsifier, gelation agent and stabilizer [2,3].

Pectins, as polymers of galacturonic acid (GalA), exert excellent binding capacity specifically towards polyvalent metal ions primarily by their chelation [4]. The use of pectin-based enterosorbents is determined by its innate ability to effectively bind heavy metals (HM), which was proven in numerous experimental studies [5,6,7,8] and in vivo [9,10].

Although acute poisoning with HM is not very common and considered as an “orphan disease” [11], a large number of people living in areas with extensive industrial activities experience chronic exposure to these xenobiotics usually by drinking contaminated water, particularly in developing countries [12].

A frequent cause of acute poisoning is a drug overdose with non-steroidal anti-inflammatory drugs (NSAID) such as diclofenac and paracetamol leading to drug-induced liver damage. It occurs more often due to suicide attempts and less commonly due to pain treatment in combination with opioids and in association of alcohol intake [13]. In the UK, the top position in etiology of fulminant liver failure occupies the NSAID-induced poisoning, which exceeds the number of cases related to viral hepatitis [14]. In the US, NSAID-related acute intoxications leading to hospitalization are registered as frequently as 29, in Israel—57 and in Great Britain—200 cases per 100,000 population annually. Whereas the mortality rate in cases of toxic hepatopathy is 5%, for NSAID intoxication (even in low doses) it reaches 19% [13]. In this regard, the problem of prevention and mitigation of drug induced acute poisoning, including such drugs as diclofenac, is relevant. With diclofenac being a relatively small/medium size organic molecule (molecular weight, MW = 296 Da), enterosorption using activated carbon could provide an efficient treatment for such patients.

Rice husk (RH) is a unique agricultural by-product in terms of its abundance; large-scale world production is estimated at over 140 million tons annually [15]. In rice growing countries, rice husk has minimal commercial value and traditionally has been burnt at rice fields. Rice husk is not readily decomposed by bacteria and causes air pollution upon incineration thus posing a significant environmental problem, which requires solutions by finding more environmentally friendly means of its utilization [16]. In rice producing countries, RH is used as a fuel. However, this product is characterized by low calorific value, 13–15 MJ/kg [17] and high ash content, most of which is composed of silica [18]. The nanoscale silica phytoliths present within the rice husk material may serve as a template to create additional pore space within the nanoscale range [18,19,20].

Converting RH into activated carbon not only could create a substantial added value to the product with its potential use in adsorption and catalysis [21,22], but also reduce the inherent problems associated with current methods of this renewable biomass waste management, offering a “green technology” approach.

Recently there has been a renewed interest in utilizing RH-based activated carbon (AC-RH) as a low-cost sorbent for removing heavy metals and organic molecules from aqueous solution, as well as for biomedical applications [23,24,25].

One of the important requirements for enterosorbents, in addition to their sufficient sorption capacity, is the absence of an irritating effect on the mucous membranes of the gastrointestinal tract and acceptable palatability [10,26,27]. The disadvantage of solid tablet forms of enterosorbents is their low affinity to the surface of the mucosa in the intestinal lumen, which sharply reduces the contact of the enterosorbent with toxins; irritating effect on the intestinal mucosa and low ability to bind and remove toxins. Many patients have problems with activated carbon intake as they consider it having low palatability. On the other hand, pectin being a natural product used in making food, has satisfactory palatability. It has been shown that pectin can be combined with other adsorbents for improved removal of hydrophilic and hydrophobic contaminants [28,29,30,31].

Combining pectin and activated carbon in a new enterosorbent formulation could lead to the design of a material capable of binding a wide range of toxic substances not achievable by individual sorbents.

In this work, we developed a microgranulated binary enterosorbent preparation with a «core-shell» structure based on low-esterified pectin as the core, with the shell being made of fine powder of nanoporous activated carbon derived from RH and studied its ability to bind xenobiotics of organic origin (diclofenac) and heavy metals (lead ions).

In the “core-shell” microgranular composition, the hydrated pectin core would not inhibit the adsorption of toxins onto the activated carbon surface in the shell during pectin swelling upon entering the gastrointestinal tract. At the same time, AC-RH microparticles should not block the migration of metal ions into the core of the low-esterified pectin gel, which binds polyvalent metal cations [4].

## 2. Results and Discussion

### 2.1. Synthetic Approach (Methodology) Insight

ACs utilization is especially designated for organic compounds, in particular containing aromatic rings, halogen and sulfide functionalities in the molecular structure, and that is primarily due to interactions caused by Van der Waals forces in the micro-mesopore interface on the surface. On the contrary, pectins, as polymers of galacturonic acid, exert excellent binding capacity specifically towards divalent metal ions primarily due to chelation in accordance with “egg-box” mechanism.

For effective elimination of a mixture of both inorganic and organic xenobiotics from the gastrointestinal tract (GIT) by means of enteral detoxification therapy, it is necessary to apply compositions of different classes of adsorbents. For example, cellulose, chitosan, pectin, etc., acting on the principle of chelation when interacting with polyvalent metal cations. On the other hand, adsorbents, such as porous activated carbon, have a high specific surface area, on which the binding of organic toxins occurs due to the Van der Waals forces. However, when a gel and an activated carbon are used together, the problem of diffusion restrictions arises. In particular, the hydrogel is able to envelop the pores of the activated carbon, thereby blocking access to the surface.

We propose to apply the approach, according to the principle of a “core-shell” hybrid composition in the form of microgranules/beads, where the core is pectin, and the shell is fine powder of nanoporous activated carbon. An innovative approach, which is able to significantly reduce diffusion restrictions, since, one hand, the hydrated pectin core physically is not able to inhibit the adsorption of toxins on the activated carbon surface during swelling upon entering the gastrointestinal tract. On the other hand, activated carbon microparticles not only partially adsorb by themselves, but at the same time do not block the migration of the excessive metal ions into the core of the low-esterified pectin gel, which effectively binds polyvalent metal cations by means of the “zip-lock”/“egg-box” mechanism [4]. 

**Scheme 1:**
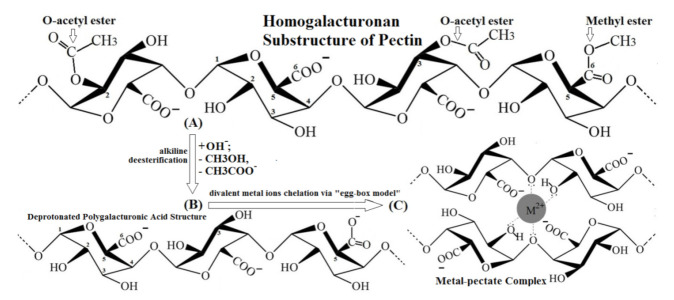
Synthesis of pectin and complex formation with heavy metals.

Homogalacturonan HG is the predominant and linear pectin domain with a backbone consisting of α-1,4-linked GalA units which can be methylesterified at their C-6 position or O-acetylated at the C-2 or C-3, as can be seen in Scheme 1.

The role of these structural properties is only briefly discussed, given that the current review is focused on pectins commercially used in pectin–cation associated functionalities. These pectins include particularly apple pectin, in which the degree of acetylation and neutral sugar side chains play a limited role as these pectins naturally exhibit a low degree of acetylation. For instance, sugar beet pectin, which is known to be highly acetylated (35% average), exhibits poor chelating capacity and lower cation affinity compared to apple pectin [4].

### 2.2. Low-Temperature Nitrogen Adsorption Studies of Rice Husk Derived Activated Carbon (AC-RH)

**Figure 1:**
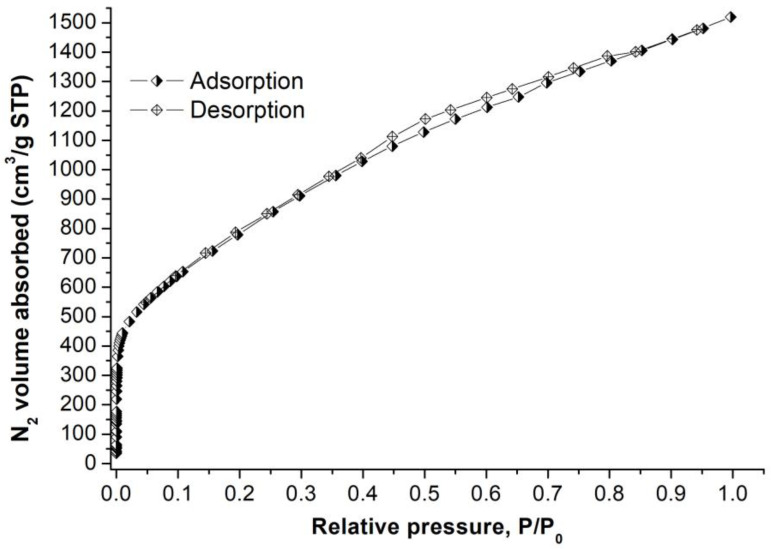
Nitrogen adsorption-desorption isotherm of the AC-RH sample.

According to the IUPAC classification, the nitrogen adsorption-desorption isotherm on the AC-RH sample (Figure 1) is an isotherm of mixed type I (b) (at low relative pressure) and type IV (at high P/P_0_) [32]. It is associated with a broad range of pore size distribution, including wider micropores and possibly narrow mesopores (<2.5 nm). The isotherm hysteresis loop is a mixture of H3 and H4 types, which indicates the presence of slit-shaped pores.

In general, activated carbon obtained from rice husk using KOH as an activating agent has a very large surface area with high meso- and micropore volumes. In our study, according to the calculations of the low-temperature nitrogen adsorption data, it was determined that the values of the specific surface area (S_BET_), DFT-surface area (S_DFT_) and the total pore volume (V_DFT_) for the AC-RH sample were 2938 m^2^/g, 2356 m^2^/g and 2.17 cm^3^/g, respectively; the values of mesopore volume (V_BJH_) and micropore volume (V_DR_) were 1.15 cm^3^/g and 1.07 cm^3^/g, respectively. S_BET_ of de-esterified sugar beet/apple (DE-SB/A) pectin and composite AC-pectin material (AC-RH@pectin) calculated by one-point BET method was 16.9 and 256.8 m^2^/g, respectively.

### 2.3. FTIR Spectroscopy and Degree of Esterification of Pectins

#### 2.3.1. FTIR Spectroscopy Qualitative Analysis

**Figure 2:**
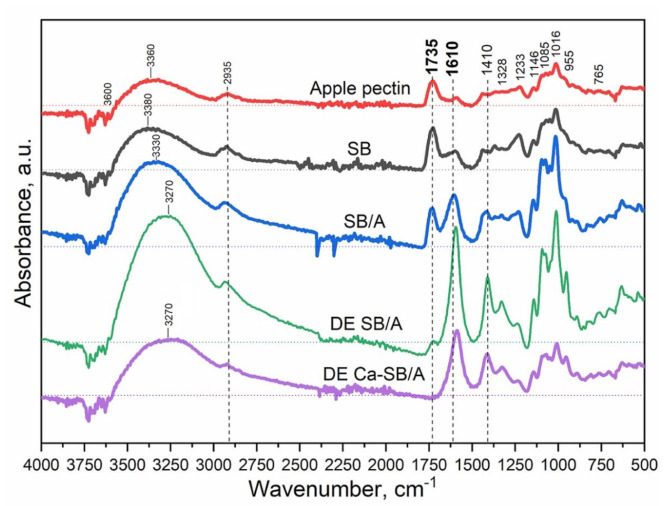
FTIR spectra of different pectins.

FTIR spectra of several types of pectin, namely: Apple, Sugar Beet (SB), mixture of Sugar Beet and Apple (SB/A), both de-esterified (DE-SB/A) and subsequently calcified (DE Ca-SB/A) pectins, used in this work are shown in Figure 2.

According to FTIR spectroscopy, the following characteristic absorption regions have been identified: (O-H) stretching vibration bands of hydroxyl groups from 3100 to 3600 cm^−1^; bands of C-H deformation vibrations (from 2800 to 3000 cm^−1^); a region that is characteristic of each type of pectin, below 2000 cm^−1^, which includes bands of the carbohydrate (galacturonic) ring (C50-) between 950 and 1200 cm^−1^; the region of carboxyl groups (-COOH) from 1200 to 1800 cm^−1^;the glycosidic bond (1146 cm^−1^), the sugar ring band in the similar range (from 990 to 1100 cm^−1^) and stretching vibrations of C-O in C-O -C and C-OH from 1016 to 1050 cm^−1^ [33].

The FTIR spectrum of acid-extracted and alkali-de-esterified pectin (Figure 2, DE-SB/A pectin) shows a change in the characteristic -COOH bands: non-ionized (esterified, or protonated) stretching peaks of -COOH at 1744 and 1637 cm^−1^ in the extracted pectin shift, after the de-esterification (demethylation or deacylation or both) process, to characteristic symmetric and asymmetric stretching vibrations -COO- at 1612 and 1406 cm^−1^. This shift of the peaks confirms the decrease in the number of methylated -COOH groups. The SB/A and DE-SB/Apectins also gelate due to acid charge compensation and are soluble in bases, which is also typical for pectins with a low content of methoxyl and acetyl groups.

Gelation caused changes in the FTIR spectra of the de-esterified pectin that correspond to the -COOH residues (Figure 2), indicating that these were the main groups involved in the process. Calcium, as a divalent metal cation, has a precipitation effect on polysaccharides that contain carboxyl groups such as pectin or alginate and can be eliminated from solution and incorporated into the gel structure in an “egg-box model” due to chelation (Scheme 1).

According to the FTIR spectroscopy data it was also found that in the process of de-esterification, as a result of hydrolysis of the ester group (-COOCH_3_), due to the loss of the methoxy group (-OCH_3_) and the formation of the hydroxyl (-OH) group, there was a significant increase in the intensity of the absorption bands of valence and deformation vibrations of (O-H) hydroxyl groups in the range from 3100 to 3600 cm^−1^ and carboxyl groups (-COOH) in the range from 1200 to 1800 cm^−1^.

There was also a shift and a change in the intensity of the absorption bands of valence vibrations characteristic of C-O in C-O-C and C-OH groups in the range from 1016 to 1050 cm^−1^.

The absorption band intensity of hydroxyl (-OH) and carboxyl (-COOH) valence vibrations decreases as a result of binding to polyvalent metals due to chelation of low-esterified pectin with divalent Ca^2+^ ions in accordance with the “egg-box model”.

#### 2.3.2. FTIR-Spectroscopy Semi-Quantitative Analysis of Pectin’s Degree of Esterification

##### Degree of Esterification (DEst)

The ratio of the area of the band at 1735 cm^−1^ (corresponding to the number of esterified carboxylic groups) [34], over the sum of the areas of the bands at 1735 cm^−1^ and 1610 cm^−1^ (corresponding to the number of total carboxylic groups) should be proportional to the degree of esterification (DEst) determined according to Equation (1) [35]:

DEst (%) = [A1735/(A1735 + A1610)] × 100(1)

where A1610 and A1735 denote the absorbance intensity, respectively, at 1610 and 1735 cm^−1^ for non-esterified (free) carboxyl groups and the esterified carboxyl groups, respectively. DEst of SB, Apple, SB/A and DE SB/A shown in Table 1.

### 2.4. Surface Structure Morphology and the Elemental Content of the Composite Enterosorbent Components According to SEM/EDS-Analysis

**Figure 3:**
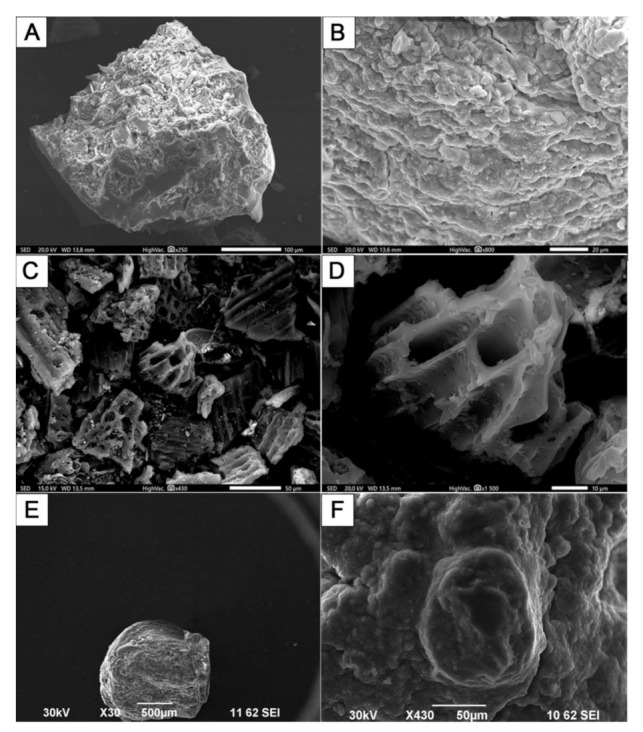
Electron microscopic images of the composite enterosorbent components: DE-SB/A pectin core (**A**,**B**) activated carbon AC-RH shell (**C**,**D**) and AC-RH@pectin composite (**E**,**F**).

The electron microscopic images (Figure 3A,B) show that the granules of the pectin xerogel are represented by a compact spongy structure with cracks. However, according to Óuwerx et al. [36], lyophilization of pectin hydrogels creates artifacts by breaking down the pore walls, and it is unlikely that the gel beads are porous when rehydrated [33].

The electron microscopic images (Figure 3C,D) show channels of porous carbon material—transport macropores; the image shows that, despite the high-temperature chemical activation with KOH, the material retained a spongy macrostructure typical of carbon sorbents based on carbonized rice husks, however, the morphology of the surface of the activated carbon AC-RH underwent a number of changes: during chemical activation erosion of the surface of the material occurred [25]. The size of the AC-RH@pectin composite microspheres was around 2 mm (Figure 3E,F).

**Figure 4:**
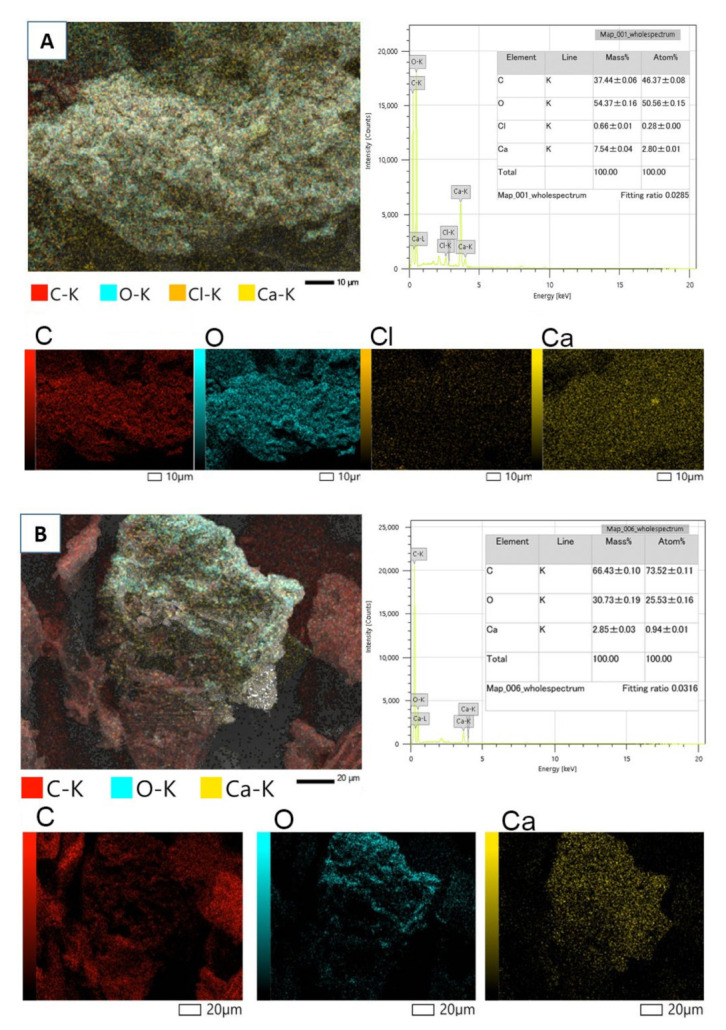
EDS-microanalysis: elemental distribution and semiquantitative elemental content of (**A**) lyophilized de-esterified pectin DE-SB/A and (**B**) lyophilized AC-RH@pectin composite.

The distribution content of elements (O, C, Ca and Cl) in the order of prevalence on the surface structure of a lyophilized de-esterified pectin core sample is shown in Figure 4A, indicating that the formation of calcium pectate took place during the sphere formation process.

According to EDS-microanalysis of the pectin core, it can be concluded that the sample mainly consists of carbon (37.44%), oxygen (54.37%) and calcium (7.54%) and only trace amount of chlorine (0.66%), which indicates the formation of calcium pectate on the surface of de-esterified pectin granules during spherification and incomplete washing out of chloride anions.

The distribution of elements (C, O, Ca) in the surface structure of AC-RH@pectin composite adsorbent is shown in Figure 4B: the distribution of elements (C, O, Ca) in the sample is uneven, with oxygen and calcium prevailing in the center of the sample, while carbon is mainly distributed along the edges of the surface. The carbon/pectin composite surface interface mainly consists of carbon (66.43%), oxygen (30.73%) and calcium (2.85%).

### 2.5. Batch Adsorption Studies

#### 2.5.1. Adsorption Kinetics

Normally, an average human stomach occupies volume of 1–2 L, which can reach the value of up to 4 L. The rationale behind the choice of lead ions initial concentration (150 mg/L) lies in the fact that the orally ingested single lethal dose of Pb^2+^ is ca. 500 mg, leading to an acute poisoning or death, if not treated timely [37]. Therefore, the model solution with initial concentration of lead ions (Pb^2+^) was set to 150 mg/L. 

In the case of the acute nonsteroidal anti-inflammatory (NSAID) drug poisoning due to an overdose, an excessive oral intake of diclofenac (DFC)can lead to multiple organ failure, such as liver injury, renal failure, CNS- disorder, cardiac arrest, and even coma or death [14,38]. In this context, the relevant sodium DFC concentration, which could cause an overdose was set at 1000 mg/L.

The pH value in the gastrointestinal tract (GIT) varies from highly acidic in the stomach (1.5–3.5) to 6 in the duodenum and small intestine, increasing up to 7.4 in the terminal ileum, decreasing again in the caecum (5.7), and reaching 6.7 in the rectum [39]. 

We used an acetate buffer with pH 5.8 and a phosphate buffer saline with pH 7.2 as simulated body fluids, in order to minimize hydrolysis during the adsorption studies.

In order to understand the kinetic behavior of Pb(II) and DFC adsorbed by the AC-pectin samples, two kinetic models: including the pseudo-first-order and pseudo-second-order were investigated, using the corresponding equations in the linear form. The pseudo-second-order model fitted the experimental data better, with relatively high coefficients of determination (R_2_ > 0.999), as compared with the pseudo-first-order model. The kinetic data of both carbons were best fitted (higher R^2^) by the pseudo-second-order model (Table 2).

**Figure 5:**
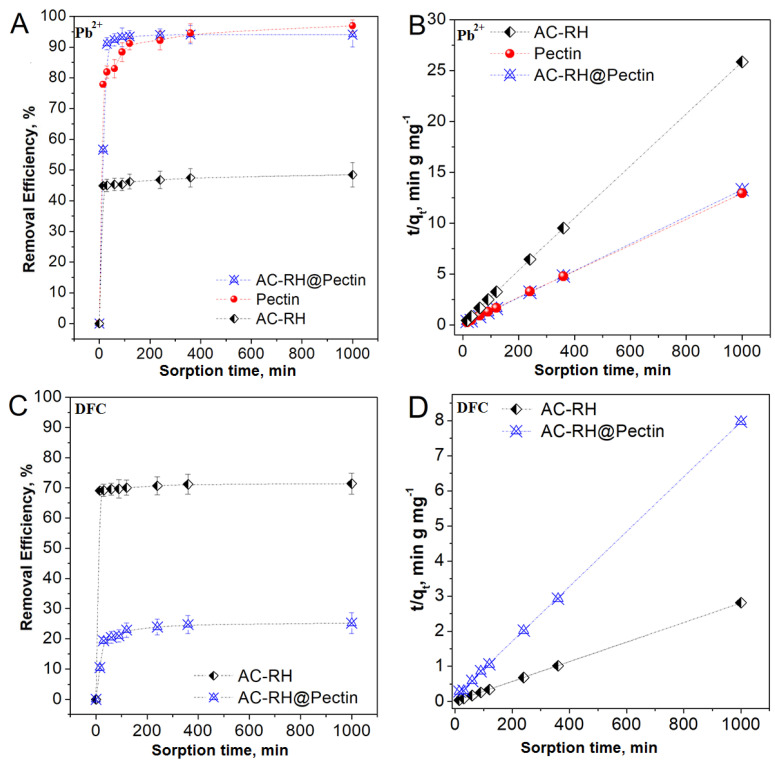
The effect of contact time on the removal rate of Pb^2+^ (**A**) and DFC (**C**) and the linearized pseudo-second order kinetic models for Pb^2+^ (**B**) and DFC (**D**) adsorbed by AC-RH, pectin and AC-RH@pectin samples. Experimental conditions: adsorbent dosage 2.0 g/L; the initial concentration and pH values are: 150 mg/L and 5.8 for Pb^2+^, and 1000 mg/L and 7.2 for DFC; temperature 310 K.

Most of lead (II) was adsorbed within 30 to 120 min (Figure 5A,C), during which the removal efficiency reached 45% for AC-RH, 80–90% for pectin and more than 90% for AC-RH@pectin samples, respectively. For DFC, the removal efficiency reached 70% for AC-RH and more than 20% for AC-RH@pectin samples, during the same contact time period.

#### 2.5.2. Adsorption Isotherms/Equilibrium Studies

**Figure 6:**
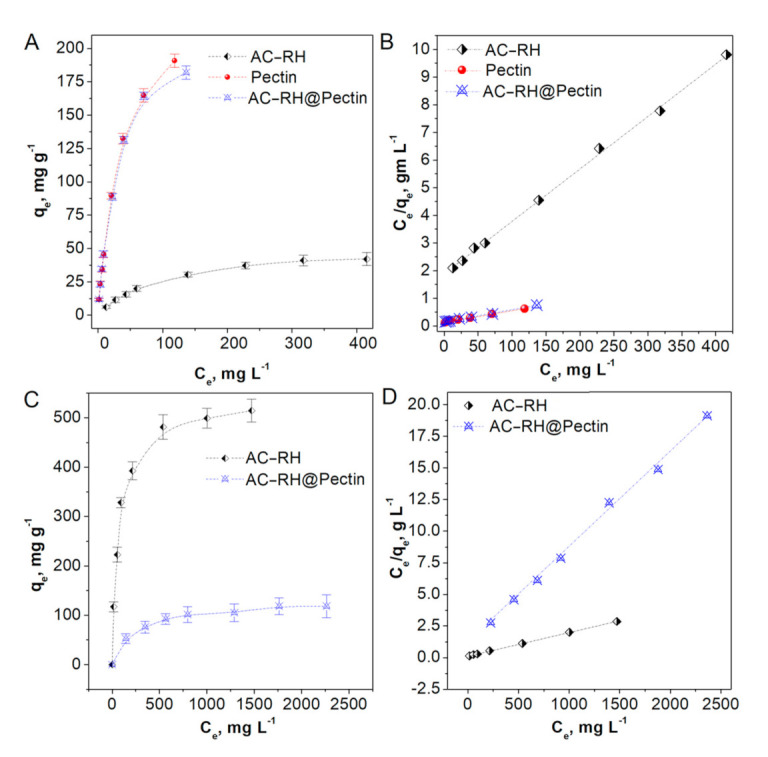
Adsorption isotherms for Pb^2+^ (**A**,**B**) and DFC (**C**,**D**) on the sorbents (AC-RH, pectin (DE SB/A) and AC-RH@pectin): experimental isotherms (**A**,**C**); linearized Langmuir model plots (**B**,**D**).

The experimental isotherms based on adsorption equilibrium study results are shown in Figure 6 as well as the fitting of Langmuir and Freundlich isotherm models. The results from isotherm modelling are summarized in Table 3. 

As a result of the data obtained using the Langmuir and Freundlich model, it was found that the maximum sorption capacity of AC-RH for DFC is 537.6 mg/g, and for AC-RH@pectin for DFC is 130.9 mg/g. Experimental isotherms are described better by the Langmuir model. 

It was found that the correlation coefficients (R^2^) of the Langmuir model (>0.992) are bigger than the R^2^ of the Freundlich model (0.879–0.981), therefore the experimental data are better represented by the Langmuir isotherm for both AC-RH and pectin, as well as AC@pectin samples in Pb^2+^ and DFC solutions.

The derived maximum adsorption capacities of AC-RH and AC-RH@pectin for DFC are comparable to the experimental values of q_max_^exp^ (Table 3), which is characteristic of relatively fast diffusion mass transfer processes and is typical for AC-adsorbents with high surface area. However, the adsorption capacity of AC-RH is nearly four times higher than that of AC-RH@pectin and in parallel with the mass ratio of AC-RH shell and pectin core.

Despite the fact that the values of correlation coefficients of the Langmuir model (0.992−0.988) are higher than those for the Langmuir model (0.963−0.981) for the Pb^2+^ solution, they differ insignificantly, suggesting a mixed type adsorption mechanism, depending on the adsorbent nature: metal ions uptake by pectin is dominated by chelation and ion exchange while AC-RH high surface operates through Van Der Waals forces, unless it is tailored with ionic functional groups. Even though AC-RH has a superior surface area (S_BET_ > 2900 m^2^/g), the value of its Langmuir monolayer capacity is comparatively low (q_max_ = 52.7 mg∙g^−1^). On the contrary, the DE SB/A pectin sample exerts rather large Langmuir q_max_ value of 245.7 mg∙g^−1^, despite much smaller S_BET_ value of 17 m^2^/g (see the Table 4). It is also necessary to note that the Pb^2+^ adsorption process is driven primarily by ion exchange process and has a diffusion limitation in the course of accessing the pectin gel active sites or AC-RH micropores. That is the reason why over 24 h adsorption time period, typical for an enterosorbent GIT residence time after administration, only a quasi- equilibrium is reached and the values of experimental adsorption capacities (q_max_^exp^) about 20% lower than that of Langmuir q_max_.

## 3. Materials and Methods

A comparison between different biomass-derived adsorbents used for DFC adsorption concerning the adsorption capacity was performed (Table 4). Although a direct comparison may be subjective due to different experimental conditions and synthesis methods, it allows having a perception about the performance of the developed enterosorbent.

### 3.1. Materials

A batch of sugar beet (SB) pulp was provided by the “Central Asian Sugar Corporation” (CASC LLP, Kazakhstan). A batch of apple pomace was purchased from a fruit canning plant (Almaty Regional Agrarian and Industrial Association, Kazakhstan). A batch of a dry mixture of sugar beet/apple pulp (mass ratio 60/40) was provided by “Technologica” Ltd. (Kyiv, Ukraine).

The analytical purity Pb(NO_3_)_2_ (≥99.0%) from Sigma-Aldrich (Germany) and the analytical purity (≥99%) sodium diclofenac (2-[(2,6-dichlorophenyl)amino]-benzeneacetic acid, monosodium salt) from Cayman Chemical (USA) were used for all adsorption experiments.

### 3.2. AC-RH@Pectin-Based Core/Shell Granular Composite Synthesis

#### 3.2.1. Activation of Carbonized Rice Husk with Potassium Hydroxide

A batch of activated carbon made from RH was prepared using the method described in detail elsewhere [25,47]. Briefly, it included three stages: (i) RH carbonization at 475 °C for 30 min to yield RH-475 sample, followed by (II) demineralization with 1.5 M HCl and (III) activation with potassium hydroxide (KOH ≥ 85.0 wt%) by using an impregnation mass ratio of 1:4 (RH-475/KOH)) at 850 °C for 2 h. After that, the sample was washed with water until pH 6–7 and dried to constant weight to yield the AC-RH sample. In order to obtain micronized fractions of the required size, the AC-RH sample was ball-milled, sieved and screened.

#### 3.2.2. Isolation of Pectin from Sugar Beet and Apple Pulp by Acidic Extraction

The pectin isolation was carried out using standard methods [48]. Some stages of pectin extraction are depicted in the photographs presented in (Figures S1–S5). The process scheme is shown in Scheme S1. In brief, sugar beet pulp and/or apple pomace was subjected to acid hydrolysis using nontoxic citric acid to extract pectins: 150 g of pulp was added to an Erlenmeyer flask equipped with a mechanical stirrer and a thermostatically controlled bath. 2 L of 1.5 wt% citric acid solution in distilled water (pH—2.5) were added into the flask. The mixture was stirred for 72 h at 60 °C (Figure S1). The resulting mixture was filtered out of cellulose, the fine filtration extract was concentrated and purified by precipitation using ethanol, followed by pectin isolation via centrifugation (Figures S2–S4). The yields of pectins were 5% for sugar beet (SB/A) pulp, 5.2% for apple pomace, and 7.2% for the mixture of (mass ratio 60/40) sugar beet/apple (SB/A) pulp, respectively.

#### 3.2.3. De-Esterification of Pectins by Alkaline Hydrolysis

De-esterification of pectins was achieved using alkaline hydrolysis followed by neutralization of alkali excess, water washing and reprecipitation with ethanol at cool temperature [46]. During this process, 10 g of high-esterified pectin was de-esterified in 150 mL 50% ethanol containing 3 g NaOH by stirring the solution at room temperature for 30 min. After titration with an equivalent amount of HCl in 50% ethanol until pH 4, the residue was filtered out, resuspended in 150 mL of distilled water and gelled using 150 mL of 96% ethanol. The de-esterified (DE) pectin was isolated from solution by centrifugation and purified by washing twice with 50% ethanol via resuspension/centrifugation to remove traces of NaCl.

#### 3.2.4. Coating of Spherized De-Esterified Pectin Beads with Micronized AC-RH

**Figure 7:**
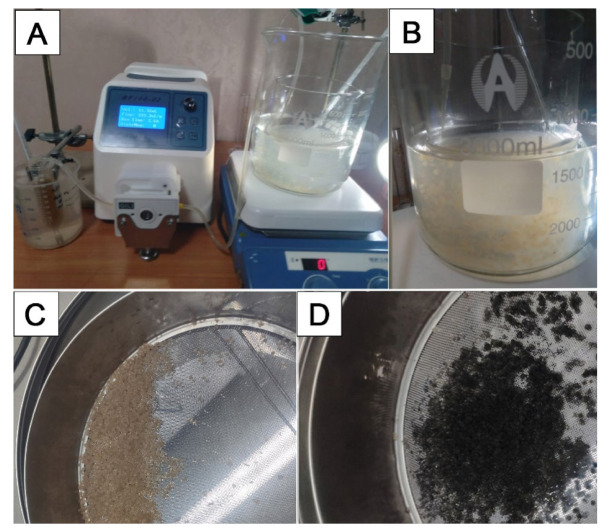
The experimental setup for pectin spherification (**A**) to yield spherical pectin hydrogel beads (**B**,**C**) covered with micronized activated carbon (**D**).

100 mL of 5% DE-SB/A pectin solution, prepared by resuspension of 5 g of DE pectin in 95 mL of distilled water, was added dropwise into a bath filled with cold (thermostated at 4–5 °C) 1.5 M CaCl_2_ solution in 50% ethanol by pumping through a capillary (inner diameter—1 mm) using a peristaltic pump (QILI BF100-02, China) at a rate of 333 μL/min [33]. The experimental setup for pectin sphere formation is shown in Figure 7. The spherical pectin hydrogel beads were kept at 4–5 °C for 12 h in the same solution. Excess of CaCl_2_ was washed with 100 mL portions of distilled water 3–5 times on a sieve (1 mm mesh), kept in 1 L of distilled water for at least 12 h at 4–5 °C and filtered. The weight of the formed spheres of pectin hydrogel beads was ca. 54.6 g.

In order to prevent sticking together, spherical pectin hydrogel beads were air blown to remove the excess of water on a sieve and dusted with a certain amount of micronized AC-RH (fraction size: 40–64 µm). The mixture was transferred into a round-bottom flask, which was then rotated on a rotary evaporator (RV 10 Basic V, IKA, Wilmington, NC, USA) at 75 rpm for 15 min at room temperature.

The content of the flask was freeze-dried using FreeZone 2.5 L (Labconco, Kansas City, MO, USA) instrument via lyophilization technique at –45 °C *in vacuo* (0.4 mbar), and then screened: first, the excess AC-RH microparticles was sieved out using a sieve (0.1 mm), then the AC-RH@pectin fraction was screened out using a sieve 2.5–3.5 mm, and the final product yield of microgranular adsorbent was 5.67 g (ca. 90%).

### 3.3. Physicochemical Characterization of Adsorbents

#### 3.3.1. Low-Temperature Nitrogen Adsorption

For low-temperature nitrogen adsorption analysis (LTNA), the AC-RH was degassed for 3 h at 200 °C. Nitrogen adsorption/desorption isotherms were recorded using an Autosorb-1 analyzer (Quantachrome, Boynton Beach, FL, USA) in the range of relative pressures from 0.005 to 0.991. The data was analyzed using “Quantachrome” data analysis software. The specific surface area S_BET_ was calculated using Brunauer-Emmett-Teller (BET) theory; the surface area (S_DFT_) and total pore volume (V_DFT_) were calculated using the density-functional theory (DFT) slit/cylindrical pore model recommended for activated carbon [49]; the mesopore volume (V_BJH_) was estimated using Barrett-Joyner-Halenda (BJH) method and the micropore volume V_DR_ was calculated using Dubinin-Radushkevich isotherm (DR) equation.

LTNA express analysis was employed to assess one-point BET surface area of pectin containing samples DE-SB/A pectin and AC-RH@pectin using Sorbtometer-M surface area analyzer (“Katakon”, Russia). These samples were first degassed at a lower temperature (80 °C) for half an hour, to avoid depolymerization.

#### 3.3.2. FTIR-Spectroscopy of Pectins

For the functional groups analysis, Fourier transform infrared spectra of fine powder pectin samples were recorded in the range of 4000–400 cm^−1^ with the resolution of 4 cm^−1^ using a Cary 600 Series FTIR spectrophotometer (Agilent Technologies) equipped with an ATR module.

#### 3.3.3. Scanning Electron Microscopy and EDS-Analysis

The morphology of adsorbents was studied with scanning electron microscopy (SEM) using JSM-IT200 and JSM-6490 LA microscopes (JEOL, Japan) at accelerating voltage of up to 20 kV: to eliminate the effects of charging, the samples were coated with thin conductive layer of gold in the vacuum chamber Quorum (Q150TES). Energy-dispersive X-ray spectroscopy (EDS-analysis) was used for a semi-quantitative assessment of the surface elemental content of the materials.

### 3.4. Batch Adsorption Experiments

For kinetic and equilibrium adsorption studies, the solutions of Pb^2+^ ions in freshly made Simulated Colonic Fluid, SCF (an acetate buffer with pH 5.8), and sodium-DFC in phosphate buffer saline, PBS, with pH 7.2 were used as dilutants, respectively, in order to inhibit hydrolysis. For SCF preparation, 10.21 g of glacial acetic acid and 6.28 g of sodium hydroxide (analytical grade) were dissolved in 1 L of distilled water [50].

The stock solutions of lead nitrate (1.589 g in 1 L) with concentration of 1 g/L of Pb^2+^ ions in freshly made SCF, and sodium-DFC (2.686 g in 1 L) with concentration of 2.5 g/L of DFC in PBS were first prepared.

Batch experiments were conducted in 50 mL Eppendorf polypropylene conical test tubes with tightly closed lids, containing 80 mg of each adsorbent (activated carbon, pectin and the resulting AC-RH@Pectin composite) and 40 mL of adsorbate solution of the required concentration, e.g., adsorbent loading dosage, defined as the solid/liquid ratio (S/L) for all experiments was fixed at 2 g/L. The solutions with added adsorbent were stirred at a rate of 150 rpm in an orbital shaker, incubated at 37 °C; at certain time points, the liquid and solid phases were separated by filtration and/or centrifugation. For the adsorption kinetics study, solutions with initial concentrations of 150 mg/L for Pb^2+^ ions and 1000 mg/L for sodium-DFC were used, respectively; and the effect of contact time in the uptake of both Pb^2+^ ions and DFC was assessed for stirring periods of 15, 30, 60, 90, 120, 240, 360 and 1000 min. For adsorption equilibrium studies, solutions with different concentrations of the adsorbate were prepared by appropriate dilution of the corresponding stock solutions, e.g.,: 25, 50, 75, 100, 200, 300, 400 and 500 mg/L for Pb^2+^ ions; and 250, 500, 750, 1000, 1500, 2000 and 2500 mg/L for sodium-DFC were used. The stirring time of 24 h was set for the adsorption experiments as sufficient to reach the equilibrium.

The adsorption capacity q, in mg/g, and removal efficiency (%) of adsorbate were calculated according to Equation (2) and Equation (3) as follows:

(2) $$q=\frac{(C_{0}-C)\times V}{m}$$

(3) $$\mathrm{Removal}\;\mathrm{Efficiency}=\frac{C_{0}-C_{}}{C_{0}}\times 100\%$$

where C_0_ and C (mg/L) are the initial and final concentrations of the adsorbate solution, respectively. V (L) is the volume of the solution (40 mL), and m (g) is the mass of the adsorbent (0.08 g). The experimental results of contact time and initial adsorbate concentration were used for adsorption kinetics and isotherm study, respectively (Table 5).

The initial and final Pb^2+^ ions solutions concentrations were determined by an atomic absorption spectrophotometer (AA-6200 instrument, Shimadzu, Japan). Properly diluted final DFC solutions concentrations of in the filtrate were determined by an UV–Vis scanning spectrophotometer (Jenway 6705, Cole-Parmer, UK) at wavelength of 276 nm, using the calibration curve obtained within the range from 0 to 85 mg/L (Figure S7). All experiments were performed in duplicate, and the results were presented as the average.

## 4. Conclusions

A composite binary adsorbent AC-RH@pectin comprising a rice husk derived activated carbon (AC-RH) and pectin with the core (pectin)-shell (AC) structure was synthesized. A method of synthesis of spherical microgranules of low-esterified pectin isolated from sugar beet and fruit pulp by extraction and de-esterification was designed. The core pectin spherical beads were coated by a shell of AC-RH powder thus creating a core-shell structure. The composite sorbent retained a spongy macrostructure, consisting of channels (transport macropores), which is characteristic of carbon sorbents obtained from rice husk. It also presented a well-developed micro-mesoporous structure created during the chemical activation of the carbonized rice husk. 

The adsorbent demonstrated effective removal of both toxic polyvalent metals (represented in this study by Pb^2+^) and xenobiotics of organic origin (represented in this study by diclofenac) from simulated body fluids. Although its capacity for either adsorbate was lower than the sorption capacity of its individual components, pectin for lead (II) and AC-RH for diclofenac per unit of mass, it retained high adsorption capacity for both adsorbates studied. These results suggest that the composite AC-pectin sorbent has a potential for use in the treatment of poisoning of different etiology as a more universal enterosorbent capable of neutralizing substances of various chemical nature by their adsorptive binding and removal from the gastrointestinal tract of patients.

## Figures and Tables

**Table 1:** Degree of esterification as function of pectin type and the maximum OH-group wavenumber.

Sample Code	SB	Apple	SB/A	DE SB/A
-OH-group maximum wavenumber, cm^−1^	3380	3360	3330	3270
DEst, %	60.3	79.1	30.4	6.5

**Table 2:** Results of pseudo-first and pseudo-second order kinetic modelling for Pb(II) and DFC adsorption.

Sample	Adsorbate	Pseudo-First Order				Pseudo-Second Order	
		q_e_^cal^ (mg/g)	*k*_1_ (min^−1^)	R^2^	q_e_^cal^ (mg/g)	*k*_2_ (g·mg^−1^·min^−1^)	R^2^
AC-RH	Pb^2+^	3.14	2.38 × 10^−3^	0.982	38.8	4.904 × 10^−3^	0.999
Pectin	Pb^2+^	11.10	3.05 × 10^−3^	0.899	77.88	1.475 × 10^−3^	0.999
AC-RH@Pectin-	Pb^2+^	3.15	6.19 × 10^−3^	0.760	75.59	4.469 × 10^−3^	0.999
AC-RH	DFC	10.46	3.32 × 10^−3^	0.902	355.9	1.655 × 10^−3^	0.999
AC-RH@Pectin	DFC	35.58	6.11 × 10^−3^	0.956	127.6	4.901 × 10^−4^	0.999

**Table 3:** Equilibrium study results: experimental adsorption capacities (q_max_^exp^), Langmuir and Freundlich isotherms parameters (adsorption time-24 h).

Samples	Adsorbate	q_max_^exp^ (mg∙g^−1^)	Langmuir Model		Freundlich Model			
			q_max_ (mg∙g^−1^)	K_L_ (L∙mg^−1^)	R^2^	1/n	K_F_ (L^1/n^∙mg^1−1/n^∙g^−1^)	R^2^
AC-RH	Pb^2+^	42.1	52.7	9.98 × 10^−3^	0.998	0.5481	1.83	0.963
Pectin	Pb^2+^	190.7	245.7	2.898 × 10^−2^	0.992	0.6483	10.66	0.981
AC-RH@Pectin	Pb^2+^	181.9	227.8	3.109 × 10^−2^	0.997	0.6424	10.35	0.966
AC-RH	DFC	514.5	537.6	1.491 × 10^−2^	0.999	0.3117	62.89	0.879
AC-RH@Pectin	DFC	118.3	130.9	4.177 × 10^−3^	0.997	0.2915	13.42	0.936

**Table 4:** DFC and Pb^2+^ adsorption capacity of different biomass-derived activated carbons and commercial ACs.

Adsorbent	Adsorbate	S_BET_, m^2^/g	Adsorption Capacity, Langmuir q_max_ (mg/g)	Reference
AC from orange peels	DFC	618	62.5	[40]
AC from cocoa shell	DFC	619	63.5	[41]
AC from potato peel waste	DFC	866	68.5	[42]
AC Calgon Filtrasorb 400	DFC	997	280	[43]
Synthetic AC “PC-1000”	DFC	1236	392.0	[44]
AC-RH (from rice husk)	DFC	2938	537.6	Present work
AC-RH@pectin composite	DFC	256.8	130.0	Present work
AC-RH (from rice husk)	Pb^2+^	2938	52.7	Present work
Citrus GENU^®^Pectin LM	Pb^2+^	2.6	120.2	[45]
DE SB pectin	Pb^2+^	N/A	129.9	[33]
DE SB/A pectin	Pb^2+^	16.9	245.7	Present work
DE citrus pectin	Pb^2+^	N/A	624.8	[46]
Pectin/AC (2:3)	Pb^2+^	344.3	279.3	[45]
AC-RH@pectin composite	Pb^2+^	256.8	227.8	Present work

N/A—not available.

**Table 5:** Kinetics and adsorption isotherm models used to study the adsorption process.

Model	Linear Equation	Parameters	Reference
Pseudo-first-order	$\ln\left(q_{e}-q_{t}\right)=\ln q_{e}-k_{1}t$	q_t_ (mg/g): adsorption capacity at a time point *t* q_e_ (mg/g): adsorption capacity at equilibrium k_1_ (min^−1^): pseudo-first-order kinetic constant	[45]
Pseudo-second-order	$\frac{t}{q_{t}}=\frac{1}{k_{2}q_{e}{}^{2}}+\frac{t}{q_{e}}$	q_t_ (mg/g): adsorption capacity at a time point *t* q_e_ (mg/g): adsorption capacity at equilibrium k_2_ (min^−1^): pseudo-second-order kinetic constant	[51]
Langmuir	$\frac{C_{e}}{q_{e}}=\frac{1}{q_{\max}K_{L}}+\frac{C_{e}}{q_{\max}}$	q_e_ (mg/g): adsorption capacity at the equilibrium C_e_ (mg/L): equilibrium concentration of adsorbate K_L_ (L/mg): Langmuir constant q_max_ (mg/g): maximum adsorption capacity of the adsorbent	[46]
Freundlich	$\log q_{e}=\log K_{F}+1/n_{F}\log C_{e}$	K_F_: Freundlich constant n_F_: adsorption intensity	[52]

## Data Availability

Not applicable.

## Supplementary Materials

The following supporting information can be downloaded at: https://www.mdpi.com/article/10.3390/molecules27072296/s1, Schema S1: Pectin isolation scheme for fruit/vegetable dry pulp; Figure S1: Hydrolysis of beet/apple pulp in 2 L of 1.5% aqueous solution of citric acid at 60 °C for 72; Figure S2: Extract concentration by (a) by evaporation; (b) gelation/precipitation of pectin using ethanol; Figure S3—Centrifugation of pectin into centrifuges (Eppendorf^®^ Centrifuge 5804) for 15 min at 7000 rpm at −4 °C followed by decantation of alcohol eluent; Figure S4: Washing of centrifuged pectin with 70% ethanol (3 times) by resuspension/centrifugation; Figure S5: Feedstock (sugar beet/apple pulp) and isolated pectin, dried in air to a constant weight; Figure S6: Appearance of a freshly obtained sample of spherified pectin on a filter in a Buchner funnel; Figure S7: UV spectrum of sodium diclofenac solution and the calibration curve.

## Sample Availability

Samples of the compounds are available from the authors.
