# Supplementary material for: Activated Carbon/Pectin Composite Enterosorbent for Human Protection from Intoxication with Xenobiotics Pb(II) and Sodium Diclofenac
Source: Molecules. 2022 Apr 1;27(7):2296. doi: 10.3390/molecules27072296 (PMC9000640; doi:10.3390/molecules27072296)
Supplement: Supplementary file 1 [file molecules-27-02296-s001.zip › molecules-1559821-supplementary.pdf]

Supplementary information

Activated Carbon/Pectin Composite Enterosorbent for Human Protection from Intoxication with Xenobiotics Pb(II) and Sodium Diclofenac

Jakpar Jandosov[1,3-5], Mo Alavijeh[2], Shynggyskhan Sultakhan[3,4], Alzhan Baimenov[3,4,8], Maria Bernardo[6], Zuiyadda Sakipova[1], Seytkhan Azat[3,4], Svitlana Lyubchyk[6], Nurzhamal Zhylybayeva[4], Gulmira Naurzbayeva[3,4], Zulkhair Mansurov[3,4], Sergey Mikhalovsky[7,9], Dmitriy Berillo[1]

[1] School of Pharmacy, Asfendiyarov Kazakh National Medical University, 94, Tole bi Street, Almaty 050000, Kazakhstan; berillo.d@kaznmu.kz

[2] Pharmidex Pharmaceutical Services, 14, Hanover Street, London, W1S 1YH, UK; mo.alavijeh@pharmidex.com

[3] Institute of Combustion Problems, 172, Bogenbay Batyr Street, Almaty, Kazakhstan; jandosov.j@kaznmu.kz

[4] Al-Farabi Kazakh National University, 71, Al-Farabi Avenue, Almaty, 050040, Kazakhstan; alzhan.baimenov@nu.edu.kz

[5] Institute of Chemical and Biological Technologies, Satbayev University, Almaty 050012, Kazakhstan

[6] LAQV/REQUIMTE, Departamento de Química (DQ), Faculdade de Ciˆencias e Tecnologia (FCT), Universidade Nova de Lisboa (UNL), 2829-516 Caparica, Portugal s.lyubchyk@fct.unl.pt

[7] ANAMAD Ltd, Sussex Innovation Centre Science Park Square, Falmer, Brighton, BN1 9SB, United Kingdom sergeymikhalovsky@gmail.com

[8] National Laboratory Astana, Nazarbayev University, 53, Kabanbay Avenue, 010000, Nur-Sultan, Kazakhstan

[9] Chuiko Institute of Surface Chemistry, National Academy of Sciences of Ukraine, 17, General Naumov Street, 03164, Kyiv, Ukraine

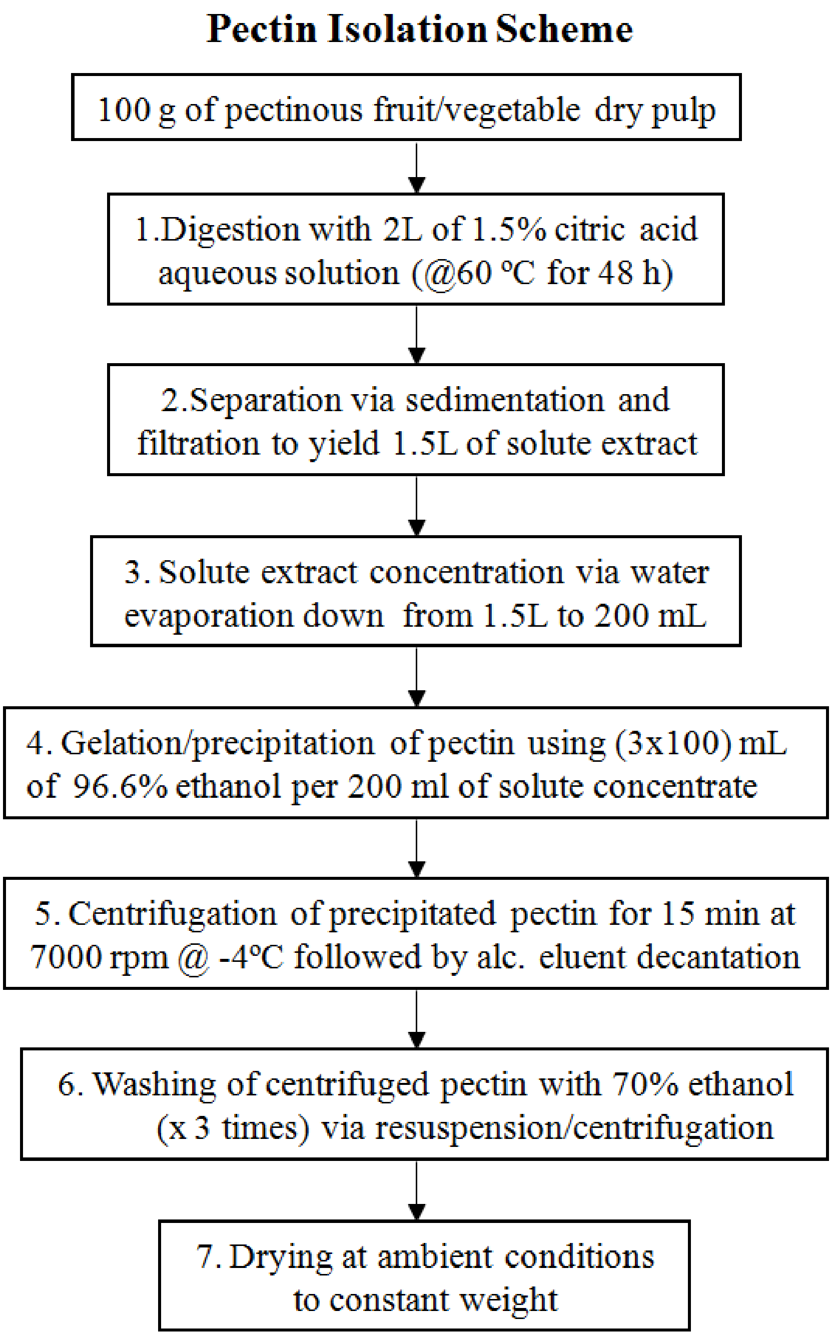

Scheme S1. Pectin isolation scheme for fruit/vegetable dry pulp

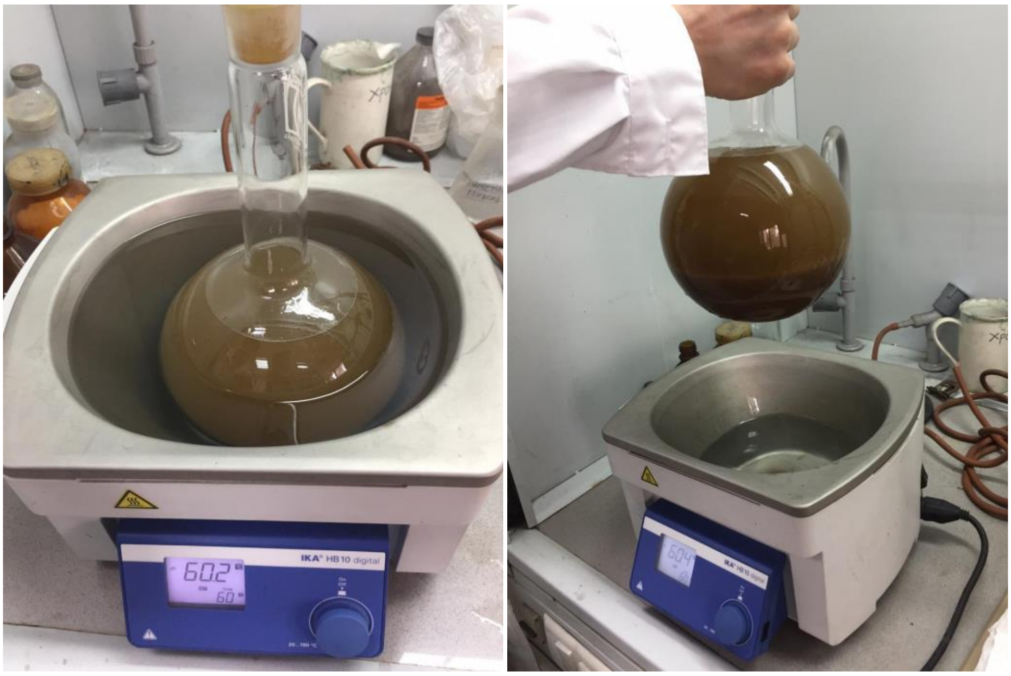

Figure S1. Hydrolysis of beet/apple pulp in 2 liters of 1.5 % aqueous solution of citric acid at 60 °C for 72

The soluble part of the extract was cooled and separated from the pulp, consisting mainly of cellulose, by sedimentation and filtration in vacuum using a Schott funnel and a Bunsen flask using a filter made of cotton towels. The filtrate (1.5 l) was evaporated to 200 ml by boiling on an electric stove in a heat-resistant glass beaker. 300 ml of 96.6 % ethanol was added to 200 ml of the cooled concentrated extract for pectin sedimentation (Figure 4).

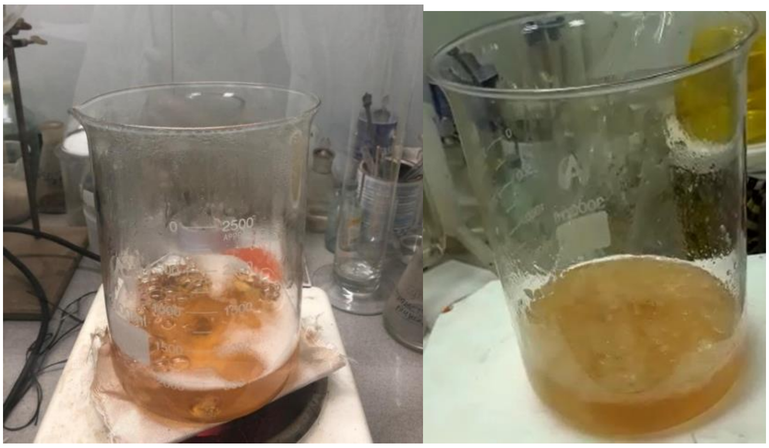

(a) (b)

Figure S2. Extract concentration by a) by evaporation; b) - gelation/precipitation of pectin using ethanol

The resulting gel-like suspension was transferred in portions to 50 ml conical Eppendorf tubes and centrifuged for 15 minutes at 7000 rpm using a centrifuge (Eppendorf ® Centrifuge 5804), thermostatically controlled at -4 °C (Figure S3). The eluent was decanted and the pectin precipitate was washed with 70 % ethanol solution 3 times 50 ml by resuspending/centrifugation (Figure S4). After air drying (Figure S5), in a drying cabinet at 60 °C to a constant weight, the mass of the isolated native pectin was 15.8 g (yield - 10.5 %).

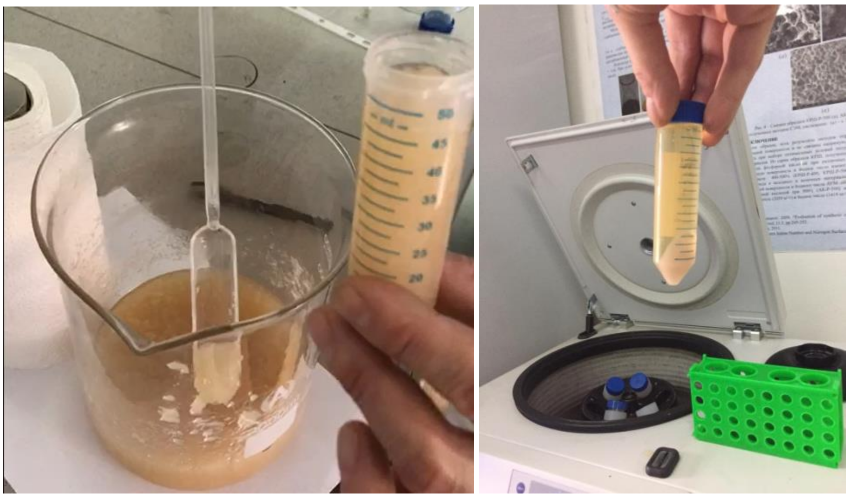

Figure S3. Centrifugation of pectin into centrifuges (Eppendorf ® Centrifuge 5804) for 15 min at 7000 rpm at - 4 ºC followed by decantation of alcohol eluent

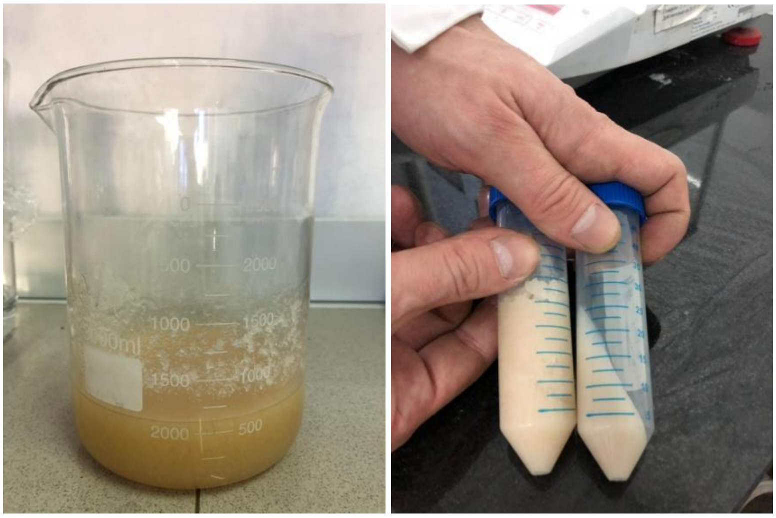

Figure S4. Washing of centrifuged pectin with 70 % ethanol (3 times) by resuspension/centrifugation

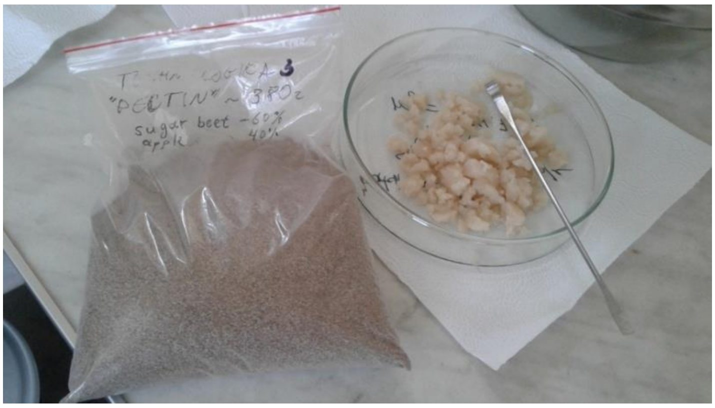

Figure S5. Feedstock (sugar beet/apple pulp) and isolated pectin, dried in air to a constant weight

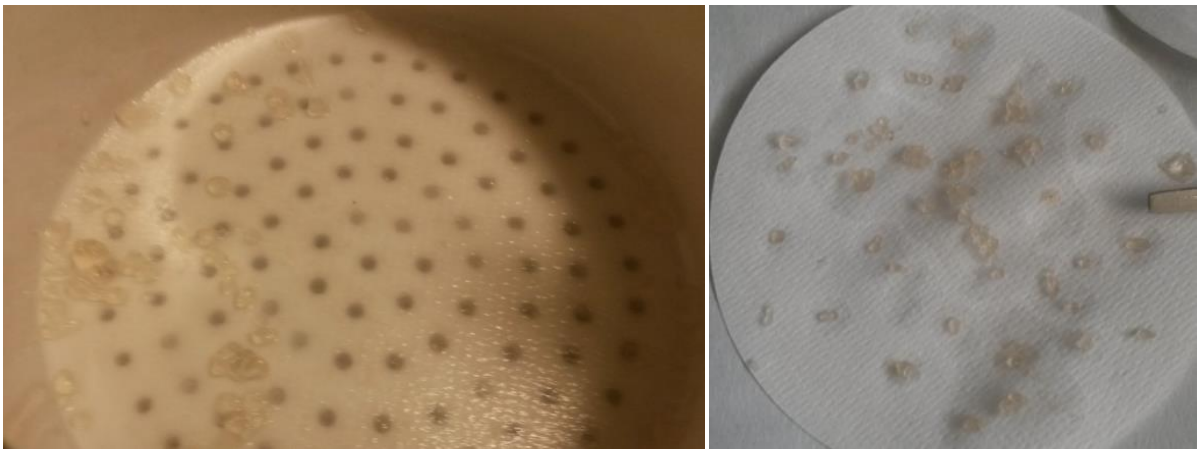

Figure S6. Appearance of a freshly obtained sample of spherified pectin on a filter in a Buchner funnel

The spherical pectin hydrogel granules were kept at 4-5 °C for 12 hours in the same solution. Excess of $CaCl_2$ was washed with portions of distilled water 100 ml × (3-5) times on a sieve (1 mm), kept in 500 ml of distilled water for at least 12 hours at 4-5 °C and filtered. The mass of the formed spherified pectin hydrogel granules was about 54.6 g.

Empirically, it was found that the optimal contact time (about 12 hours) of pectin hydrogel granules with a solution of calcium chloride leads to the formation of a strong but plastic shell of calcium pectate, but not to complete/through calcination of pectin granules.

Carbonization/steam-gas activation of the RH was carried out at 475 °C in a rotating steel reactor in a pre-assembled installation equipped with a water supply capillary, a thermocouple, the temperature rise to 475 °C was maintained at a rate of 11 °C/min; The water supply rate was set at 100 ml/hour using a peristaltic pump. The yield of the resulting CRH-475 was 38 %.

In previous studies, it was shown that in order to convert insoluble calcium and magnesium silicates into soluble chlorides, CRH-475 must be demineralized by boiling in a 5 % HCl solution. The chemistry of reactions is expressed by the following equation

$$\{Ca, Mg\}SiO_3 + 2HCl \rightarrow \{Ca, Mg\}Cl_2 + H_2SiO_3\downarrow \qquad (1)$$

At the same time, silicic acid is removed during further alkaline activation of CRH-475 at 850 ^oC using potassium hydroxide. The chemistry of reactions occurring in the process of alkaline activation of CRH can be represented by the following equations:

$$2KOH + SiO_2 \rightarrow K_2SiO_3 + H_2O \qquad (2)$$
$$H_2O + C \rightarrow CO\uparrow + H_2\uparrow \qquad (3)$$
$$2KOH + 2C \rightarrow 2K + 2CO\uparrow + H_2\uparrow \qquad (4)$$

At the same time, the formation of a porous space in sorbents obtained from RH by alkaline activation occurs due to the following processes:

1) macropores – during carbonization, due to the preservation of the native structure of the RH and the subsequent leaching of silica phytoliths from the CRH;

2) mesopore – mainly due to high-temperature leaching (desilication) of the SiO2 matrix (equation 2), as well as the carbon matrix burn off (equations 3-4);

3) micropores – mainly due to reactions of carbothermic reduction of potassium (equation 4), as well as SiO_2 leaching (equation 2) and burn off (equation 3).

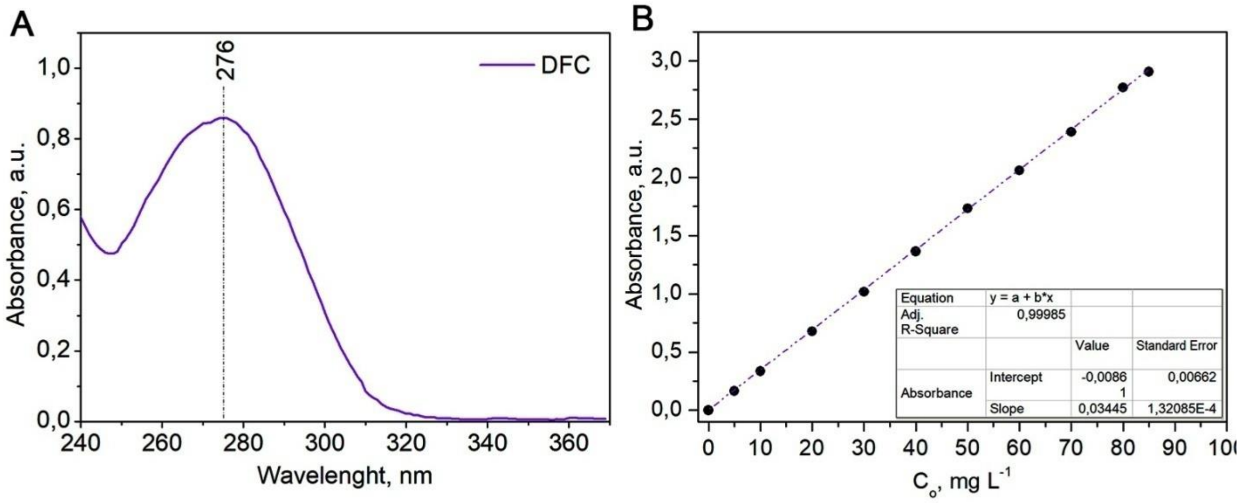

Figure S7. UV spectrum of sodium diclofenac solution and the calibration curve
